# The reasons for its effectiveness, however, remain in dispute—A tribute to Irving Kirsch

**DOI:** 10.3389/fpsyg.2022.1037678

**Published:** 2022-11-14

**Authors:** Jens Gaab

**Affiliations:** Clinical Psychology and Psychotherapy, Faculty of Psychology, University of Basel, Basel, Switzerland

**Keywords:** placebo, psychotherapy, ethics, operant conditioning, power

## Abstract

Irving Kirsch’s work spans over four decades and provided science and clinical practice with as much invaluable insights in the inner workings of treatments as it provided us and patients with their rights and our duties. Here, two early publications of Irving Kirsch on the topic of psychotherapy and its relation to placebo are revised and put into both a historical and contemporary context to pay tribute to the work of Irving Kirsch.

## Introduction

1784 might not have been a year to remember for Franz Anton Mesmer, but rather an annus horribilis. Not only were his methods and underlying principles of animal magnetism disproven by a commission of five members of the Royal Academy of Sciences, which was appointed by Louis XVI under the lead of Franklin (Franklin et al., 2002/1784), and thus his quest to obtain approval for his approach by the French government all in shambles (Donaldson, 2005), he further “suffered one of the worst humiliations of his life” when “(a) ll eyes turned toward (him) who had been unwise enough to come to the concert” of Maria Theresia Paradis in September 1784, the very pianist whose blindness he failed to heal some years before during his time in Vienna (cited from Steptoe, 1986, p. 254). Reportedly, Maria Theresia Paradis played the B Flat Concerto Köchelverzeichnis 456, which Wolfgang Amadeus Mozart wrote for her tour in Paris. However, Mozart not only provided the music to Mesmer’s social downfall, but further ridiculed the man and his methods in his opera *Così fan tutte*, in which a fake suicide is mockingly cured by “A piece of magnet, the stone which the great Doctor Mesmer discovered in Germany and then became so famous in France” (Mozart, cited from the Libretto, retrieved 8.8.2022). The families of Mozart and Mesmer were well acquainted, if not to be considered “old friends” (cited from Steptoe, 1986, p. 249) during their time in Vienna, but when Mesmer left Vienna for Paris (and his wife in misery), Mozart had little sympathy for the “errant physician, (…) after he had renounced his family in favor of the profits to be found in Paris” (cited from Steptoe, 1986, p. 255). Bereft of his academic and societal credibility, Mesmer’s “last twenty years were spent in obscurity” (cited from Steptoe, 1986, p. 255), which meant that he returned to Switzerland and Germany, where he died in 1815.

It might have been only a little gratifying for Mesmer that his work, or at least the “most important of the three causes that (Franklin and Colleagues) have (…) assigned to magnetism” (cited from Franklin et al., 2002/1784, p. 360), has found its way back in clinical mainstream, with no other than the founding fathers of neurology and psychotherapy, Jean-Martin Charcot and Sigmund Freud, making use of “this terrible, active power that produces the great effects one observes with astonishment” (cited from Franklin et al., 2002/1784, p. 360). This power at hand was nothing more than imagination, induced in the close and friendly relationship between patient and therapist. Interestingly, the power of imagination was not disputed by Charles D’Eslon—one of the last remaining truthful disciples of Mesmer and, in fact, the test subject of the Royal Commission—as “he (…) remarked to the Commissioners that the imagination directed in this way toward the relief of human suffering, would be a great blessing in the practice of Medicine” (cited from Franklin et al., 2002/1784, p. 360) or, as he put it some years before, “if medicine of imagination was the best, why would not we be doing the Medicine of the imagination?” (D’Eslon, 1780, cited as footnote in Franklin et al., 2002/1784, p. 360/361).

Clinical science has progressed substantially since the late eighteenth century, and incidentally, Franz Anton Mesmer could be considered as the unwitting originator of some of its most important developments. Not only did he and this work inadvertently give rise to what later has to become psychotherapy (Crabtree, 1994), the methods used by Franklin and colleagues to test (and disprove) Mesmer’s Animal magnetism could well be considered as the first clinical application of the principles that underlie the so-called gold standard in clinical testing: the double-blind, placebo-controlled trial (McNally, 1999; Jones and Podolsky, 2015). Furthermore, the case of animal magnetism and its disaffirmation can be considered as an early implementation of what now constitutes moral obligations in clinical ethics, as this intervention has not been condemned due to a lack of effectiveness—the intervention had notable effects!—but rather because it could not prove its assumed mechanisms, but pose a threat to patients as “the touchings, the repeated action of the imagination in producing crises can be dangerous; and that, (…) can in the long run have only disastrous effects” (cited in Franklin et al., 2002/1784, p. 362). With regard to psychotherapy, it has been noted that “despite the many similarities between the history of Mesmerism and the history of EMDR, there is at least one important difference. A prestigious committee of scientists concluded that the effects of Mesmer’s therapy were attributable to the power of suggestion, not the power of “animal magnetism,” thereby discrediting the Mesmerism movement. In contrast, the American Psychological Association’s (APA) Committee on empirically validated treatments recently startled many psychologists by proclaiming Eye Movement Desensitization and Reprocessing (EMDR) as “probably efficacious for civilian PTSD” (…) because it was statistically superior to no treatment at all in two controlled trials. Had Franklin and Lavoisier applied these criteria, they might have arrived at similar conclusions about the “probable efficacy” of animal magnetism therapy” (cited from McNally, 1999, p. 235). Thus, not all that glitters are gold and it is more than fitting that sanocrysin, a gold compound which was called “A Gold Cure for Tuberculosis” in the (Editorial of the American Journal of Public Health, 1925) in 1925, was the first treatment that was tried and tested against placebo (Burns Amberson et al., 1931). Interestingly, this first and in the end negative, test against placebo was not only driven by clinical, but also by commercial interest (Gabriel, 2014)—a bias still observable today (Lundh et al., 2017).

The previous historical anecdote provides many stepping stones to ponder on Irving Kirsch and his work. Next to both Irving Kirsch’s, as well as Franz Anton Mesmer’s, musical aptitude—the former allegedly played the violin well enough to accompany Aretha Franklin (Davies, 2014), the latter played the glass harmonica good enough to be commended by Leopold Mozart (1773), cited as footnote in Steptoe, 1986, p. 253)—both have employed suggestibility clinically, only with the very important distinction of doing this advertently open (Kirsch, 1994) or non-advertently deceptive (see Franklin et al., 2002/1784). And maybe it is this dialectic ability to acknowledge an intervention’s effect while still questioning its mechanisms, and thus its credibility, that is not only a defining, but also a distinctive feature of Irving Kirsch’s work. This can easily be traced in his seminal work on antidepressants as much as in his scientific scrutiny in placebo research, but here, this shall be exemplified in his work on psychotherapy on the basis of the two selected articles, which were published some 40 years.

In winter of 1974, Irving Kirsch must have been just about to finish his PhD at the University of Southern California when he published an article in “Psychotherapy: Theory, Research and Practice” which, in retrospect, might not only have been a harbinger for what was to come from this then promising young researcher, but which also constitutes a very fine example of why effectiveness is not the sole and last justification for an intervention and thus needs to be sided by ethics. In this article, Irving Kirsch pointedly carved out the atrocities caused by operant conditioning program in Vietnam in the late 1960s as well as the hypocritical attempts to whitewash this misuse of behavior therapy by then prominent proponents, who claimed that what has been labeled as operant conditioning was, in fact, nothing but coercion. This argument was countered not only by the comprehensive knowledge of the then existing literature on this matter—contracting the same authors at hand by quoting from his own publications—but also further substantiated by a thorough analysis of behavioral programs in American hospitals. For example, the case of “Wilma” was reviewed. This 57-year-old patient had multiple somatic symptoms and often complained about them. This behavior was seen as problematic and thus targeted by extinction through being ignored by staff. While this approach had the desired outcome, Irving Kirsch points out the “question of whose problem it is that the therapy is designed to solve” as “(Wilma) did not come to the hospital stating ‘I complain too much”’ (cited from Kirsch, 1974, p. 313). Noteworthy, he not only refuted this opportunistic and inconsiderate use of operant procedures for ethical reasons, but also criticized it from a learning theory perspective as the patient “Wilma” will be sent “back into society minus her disturbing (to others) behavior, the system which generated the disturbing behavior is left intact.” This empirical, ethical, and theoretical rebuttal is then followed by a provocative analysis of the economic and societal pressures in the hospital setting, in which patients are seen “(a)t the bottom of the hospital hierarchy (…) expected to produce socially acceptable behavior,” whereas “(the) primary function (of psychologists) is to increase the efficiency of production of socially acceptable behavior” (cited from Kirsch, 1974, p. 315). Reading this article nearly 50 years after its publication, one might not only be astounded by the courage and the academic articulation of young Irving Kirsch, but also by how much this is needed for the “(b)alance of power” (cited from Kirsch, 1974, p. 314). Psychological theories and methods are far from being innocuous and benign, with the case of so-called “enhanced interrogation” being a clear example (Hoffman et al., 2015; Seligman, 2018), and thus, this first article by Irving Kirsch might serve as a constant reminder of how this could be done and why this is necessary.

Having established himself as an assistant professor at the University of Connecticut, in 1978 Irving Kirsch addressed another issue in psychotherapy, which started as a promise but turned out to be a problem or in his own words: “(W)hile a scientific revolution may resolve many perplexing problems, it also makes many prior solutions newly problematic” (cited from Kirsch, 1978, p. 255, with reference to Kuhn, 1970). The underlying question here was how to establish efficacy in psychotherapy, which would not only inform patients, therapists, and the society at large about whether, when, and for whom psychotherapy works, but could also serve as a starting point to elucidate what really matters, that is, how psychotherapy’s often impressive effects are brought about. But even though Rosenzweig (1936) some 90 years ago should have ended the quest for supremacy of one approach when he introduced psychotherapy scholar’s sword of Damocles with his so-called Dodo verdict, the “horse race” (Beitman, 2004) in psychotherapy research is still the prevailing paradigm as much as still a matter of debate (Wampold et al., 1997; Marcus et al., 2014).

Specificity of given treatment is not elucidated by comparisons between different treatment, but by placebo-controlled trials. However, this is problematic in psychotherapy research as it is riddled by the lack of a methodologically sound comparator. This is where Irving Kirsch in 1978 sets in. There, he not only picks the then (and still!) popular concept of a general and non-specific placebo apart, but also describes the process of how “unlike a rose, a placebo by another name smells much sweeter,” that is, how “yesterday’s placebo sometimes does become today’s treatment” (cited from Kirsch, 1978, p. 257). This upcycling, from previous control conditions to accepted evidence-based treatment, is a somewhat neglected phenomena, even though (or maybe because) it affects well-known interventions, such as interpersonal therapy (Weissman, 2006). The argument used by Irving Kirsch is that if placebos are understood as treatments that are not currently understood, then we would need to consider psychotherapy itself as a placebo. He illustrated this on the example of systematic desensitization, which explanatory odyssey included reciprocal inhibition, non-reinforced exposure, and operant desensitization. This position was supported by McGlynn et al. (1981) 3 years later who stated that “(t)he state of theory in desensitization is confusing. The research done heretofore is unimpressive” (cited from McGlynn et al., 1981, p. 149), which in turn might have contributed to the decline of academic interest in this treatment (McGlynn et al., 2004). Furthermore, Kirsch argued that if a placebo is defined by the lack of an agreed-upon theory, how would you differentiate between placebo, rational emotive therapy and cognitive therapy, as they are all based on altering client expectancies? Thus, the promise, that is, the advent of cognitive therapies, became a problem because the previous conception of placebo as a treatment that “alter(ed) beliefs, expectancies, or feelings of hope and confidence” were “central to cognitive-behavioral perspectives” (cited from Kirsch, 1978, p. 260). Placebos are not mere drivers of a general expectancy of improvement, but something more, or as Irving Kirsch elegantly put it: “If this were all that were necessary to obtain a placebo effect, it would not be necessary to administer the treatment that has been described” (cited from Kirsch, 1978, p. 261).

Irving Kirsch concluded with a number of statements that must have been provocative at the time as they seem clairvoyant now as they have been substantiated by clinical trials and meta-analyses since then. First, Irving Kirsch questioned that “we do not seem to have developed any therapeutic technique that has been convincingly demonstrated to be effective beyond its ‘placebo’ characteristics” (cited from Kirsch, 1978, p. 263). This only superficially resembles Eysenck’s (1952, 1994) controversial “psychotherapy is placebo” claim as Kirsch does not use the placebo argument in favor of behavioral therapy, but to psychotherapy in general. This appears like a sweeping blow, but consider that different methods, approaches, and techniques psychotherapy do not statistically and/or clinically surpass placebo conditions (Baskin et al., 2003; Cuijpers et al., 2012) or even placebo pills (Cuijpers et al., 2014). Considering the often more than impressive effects of placebos and “negative placebos” (the word nocebo already existed (Kennedy, 1961), but apparently was not in common use), Kirsch then argued that “it seems to me that we may be on the wrong track when we attempt to control for this factor and to focus on what remains when it is removed” (cited from Kirsch, 1978, p. 263). This statement was based on the finding that differences between systematic desensitization and control conditions were mostly the result of the fashioning of respective control conditions. But considering that so-called placebo conditions in psychotherapy are often intentionally handicapped by topic restrictions and structural disadvantages and often end up as what Kirsch and others have listed in 2016 as leisure reading; and answering questions or talking about hobbies, newspapers, magazines, favorite foods, favorite sports teams, daily events, family activities, football, vacation activities, pets, hobbies, books, movies, and TV shows (cited from Kirsch et al., 2015, p. 122–123), this strong statement still holds true today and much of psychotherapy’s “tilting of windmills” (cited from Kirsch et al., 2015, p. 121) would have been spared if this call had been heeded.

It can be assumed that the powers Franz Anton Mesmer appropriated and used to his own benefit were the same as those examined by Irving Kirsch during his impressive career “to specify the laws governing its operation, to devise means of reliably producing it and of maximizing its impact” (cited from Kirsch, 1978, p. 263). However, whereas Mesmer’s work was rightfully pilloried by Mozart, one can only pay tribute to the work of Irving Kirsch. Psychotherapy is effective, but “the reasons for its effectiveness, however, remain in dispute” (cited from Kirsch and Henry, 1977, p. 1,052). Because of him and his work, we are better equipped to change this.

## Author contributions

The author confirms being the sole contributor of this work and has approved it for publication.
